# *Enterococcus faecalis* persists and replicates intracellularly within neutrophils

**DOI:** 10.1128/iai.00364-25

**Published:** 2025-12-16

**Authors:** Claudia J. Stocks, Ronni A. G. da Silva, Haris Antypas, Navin Jeyabalan, Siu Ling Wong, Kimberly A. Kline

**Affiliations:** 1 Singapore Centre for Environmental Life Sciences Engineering, Nanyang Technological University 54761, Singapore, Singapore; 2 Institute for Molecular Bioscience, The University of Queensland, Institute for Molecular Bioscience 1974, Brisbane, Australia; 3 Antimicrobial Drug Resistance Interdisciplinary Research Group, Singapore-MIT Alliance for Research and Technology 208287, Singapore, Singapore; 4 Lee Kong Chian School of Medicine, Nanyang Technological University 54761, Singapore, Singapore; 5 Tan Tock Seng Hospital 63703, Singapore, Singapore; 6 Department of Microbiology and Molecular Medicine, Faculty of Medicine, University of Geneva 27212, Geneva, Switzerland; University of Pennsylvania Perelman School of Medicine, Philadelphia, Pennsylvania, USA

**Keywords:** wound infection, host-pathogen, neutrophils, *Enterococcus faecalis*

## Abstract

Chronic wound infection is a major global public health issue, with *Enterococcus faecalis* among the most commonly isolated pathogens from such wounds. Neutrophils are short-lived immune cells critical for host defense, yet *E. faecalis*–neutrophil interactions are poorly understood. Here, we show that instead of eliminating *E. faecalis*, neutrophils provide a niche for intracellular persistence and replication, potentially prolonging infection and inflammation at the wound site. In murine wound beds and *ex vivo* wound cells, intracellular *E. faecalis* was detected in recruited neutrophils at 24 h post-infection (h p.i). Unexpectedly, extended infection did not induce neutrophil death. Rather, *E. faecalis* infection significantly prolonged the life spans of both murine and human neutrophils *in vitro* compared to uninfected controls. Quantification of intracellular CFU revealed that *E. faecalis* were phagocytosed regardless of opsonization and persisted intracellularly up to 24 h p.i. This finding was confirmed via transmission electron microscopy and confocal microscopy. Blinded quantification and fluorescent D-amino acid staining, which marks newly synthesized bacterial peptidoglycan, revealed active replication within murine neutrophils between 6 and 18 h p.i., followed by a predominately persistent phase between 18 and 24 h p.i. Infected murine neutrophils remained immunologically active, secreting pro-inflammatory and chemoattractant cytokines. These findings highlight an underappreciated intracellular lifestyle for *E. faecalis* that may contribute to its ability to persist in chronic wounds and contribute to biofilm-associated infections.

## INTRODUCTION

Chronic wound infection represents a major global public health concern, impacting both healthcare costs and patient quality of life (1, 2). *Enterococcus faecalis* is a Gram-positive opportunistic pathogen associated with infections in a range of contexts, including urinary tract infection, endocarditis, neonatal sepsis, and chronic wound infection (3–7). A facultative anaerobic bacterium and commensal of the human gastrointestinal tract, *E. faecalis* exhibits both intrinsic and acquired antibiotic resistance (8), making these infections inherently and increasingly difficult to treat. Long considered an extracellular pathogen, *E. faecalis* aggregates and forms biofilms, enhancing its persistence capacity in chronic infections (9). However, increasing evidence across a range of host cell types points to an intracellular niche and lifestyle of this multifaceted persistent bacterium (10–13).

Neutrophils are short-lived, highly antimicrobial cells of the innate immune system that are among the first cells to be recruited to sites of infection (14). Bacterial phagocytosis typically triggers neutrophil death (apoptosis) (15–18); however, some intracellular pathogens can subvert this process to persist or proliferate within neutrophils (19–22). Research on the interactions between *E. faecalis* and neutrophils is scarce. Existing studies, all >25 years old, focused on early stages of the interaction (10–120 min), including attachment (23), phagocytosis (24, 25), and rapid killing (26–28). These reports identified the importance of serum opsonization for effective neutrophil killing of *E. faecalis* (26, 27). In the absence of serum opsonization, substantial uptake (but not killing) of *E. faecalis* expressing aggregation substance was reported in neutrophils (24, 28) and macrophages (29). Overall, these studies have led to the assumption that neutrophils effectively eliminate *E. faecalis*.

In a mouse model of wound infection, we previously observed *E. faecalis* microcolonies in the wound bed, together with a pro-inflammatory cytokine response and an influx of polymorphonuclear leukocytes (30), similar to that observed with cutaneous *Staphylococcus aureus* (31) and *Pseudomonas aeruginosa* infections (32). Despite this robust acute inflammatory response, *E. faecalis* can persist in murine wounds for at least 7 days (30). This discrepancy between *in vitro* evidence of rapid neutrophil clearance and long-term *in vivo* persistence presents a knowledge gap in our understanding of *E. faecalis*-neutrophil interactions (7).

Here, we investigated the neutrophil response to *E. faecalis* following the observation of clusters of *E. faecalis* within neutrophils in murine wound tissue at 24 h post-infection (p.i.). Examining neutrophil*–E. faecalis* interactions *in vitro*, we unexpectedly found that phagocytosed *E. faecalis* infection suppressed, rather than induced, neutrophil death in both mouse and human cells up to 24 h. Transmission electron microscopy showed *E. faecalis* residing within spacious vacuoles inside neutrophils. Intracellular quantification and the use of a fluorescent dye to mark newly formed bacterial cell walls revealed that *E. faecalis* was not only persisting but replicating within murine neutrophils. Multiplex cytokine analysis of *E. faecalis*-infected murine neutrophils identified the release of pro-inflammatory cytokines to signal for macrophage recruitment. These findings challenge the prevailing view of neutrophils as fully effective against *E. faecalis* and further our growing appreciation of its capacity for intracellular replication.

## RESULTS

### *E. faecalis* **i**ntracellular clusters are present within neutrophils during wound infection

**Fig 1:**
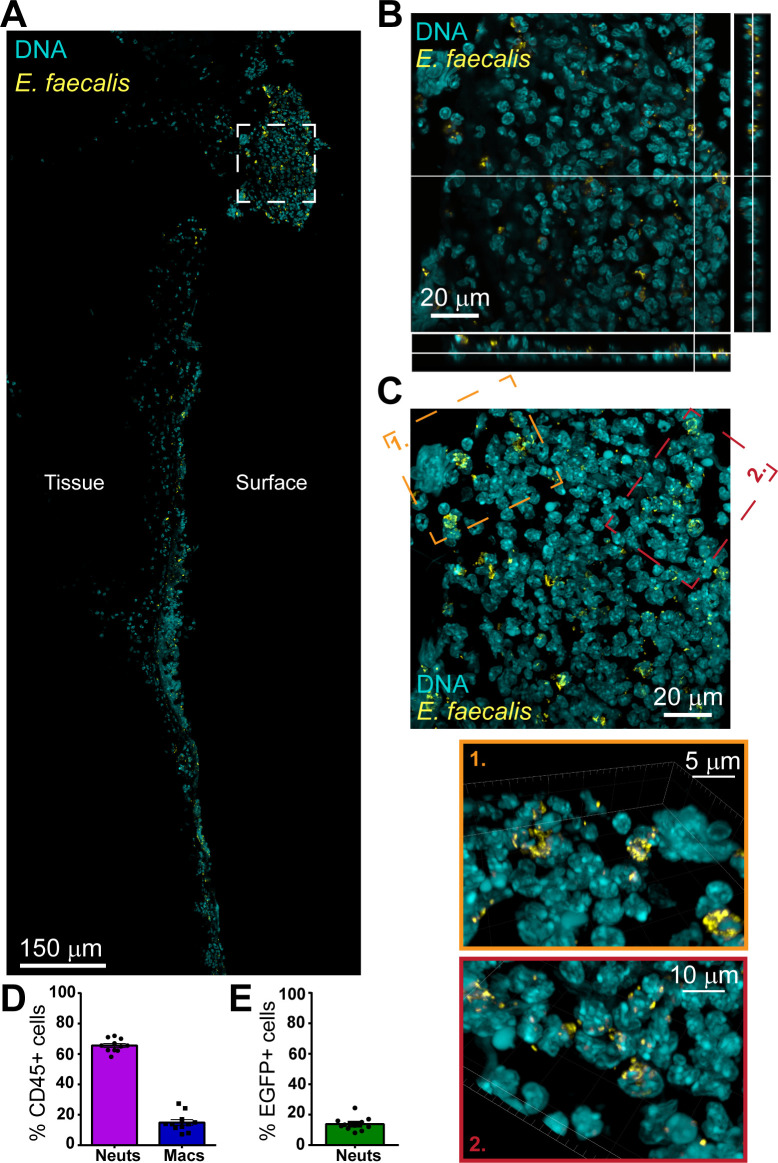
Intracellular clusters of *E. faecalis* within neutrophils in the murine wound bed at 24 h p.i. Mice were wounded and infected with 10^6^ CFU of *E. faecalis* OG1RF (**A–C**) or OG1RF pDasher (**D, E**) for 24 h. (**A–C**) Wounds were excised, fixed, and embedded before being cryosectioned, mounted on slides, and stained for Streptococcal Group D antigen to mark *E. faecalis* bacteria (yellow) and DAPI for DNA (cyan). Sections were imaged at 63× on a confocal microscope. (**A**) Depicts a tile scan of a portion of the entire wound bed, with tissue and surface sides indicated. The white inset box is shown in (**B**) as a single slice from the z stack with ortho view and in (**C**) as a max intensity projection, with additional zoomed-in angled orange and red insets of IMARIS 3D reconstructions. (**A–C**) Are from a single experiment and mouse, representative of *N* = 6 mice from three independent experiments. (**D–E**) Wounds were excised, and then cells were dissociated from this tissue via liberase reagent. Cells were then stained and imaged via a BD LSRFortessa X-20 Cell Analyzer. (**D**) Displays the relative proportion of (Ly6G^high^, CD11b^+^) neutrophils compared to (Ly6G^low^, F4/80^+^, CD11b^+^) macrophages in the CD45 + immune cells analyzed. (**E**) Further displays the proportion of neutrophils that were EGFP (i.e., *E. faecalis* OG1RF pDasher-positive). (**D, E**) Depict mean + SEM from *N* = 6 mice across three independent experiments.

To investigate the mechanisms underlying *E. faecalis* persistence in wounds, we visualized and characterized its localization *in vivo* using our established mouse wound infection model (30). Wounds were inoculated with 10^6^ CFU *E. faecalis* OG1RF for 24 h, then cryosectioned and immunostained for Streptococcal Group D Antigen to detect *E. faecalis* before imaging via confocal microscopy. Tile scanning revealed widespread *E. faecalis* clusters throughout the wound bed (Fig. 1A), including both extracellular microcolonies and apparent intracellular clusters (Fig. 1B and C). This observation is consistent with previous reports of viable *E. faecalis* within both CD45^+^ (immune) and CD45^−^ (non-immune) wound cell populations at 1–3 days p.i. (12).

To determine host cell types containing intracellular *E. faecalis*, we repeated the wound infection using a GFP-fluorescent strain of OG1RF, *E. faecalis* pDasher (33). Single cells were isolated from infected wounds and stained for immune cell markers (CD45^+^) to discriminate between neutrophils (Ly6G^high^, CD11b^+^) and macrophages (Ly6G^low^, F4/80^+^, CD11b^+^) (Fig. S1A). Of the immune cells detected, ~70% were neutrophils, and ~15% were macrophages (Fig. 1D). Among the neutrophils, ~15% were GFP^+^, indicating the presence of *E. faecalis* pDasher (Fig. 1E; Fig. S1B). Given the likely pDasher plasmid loss during infection, this is probably an underestimation. These findings demonstrate that *E. faecalis* clusters reside intracellularly within neutrophils during wound infection.

### *E. faecalis* suppresses neutrophil death

**Fig 2:**
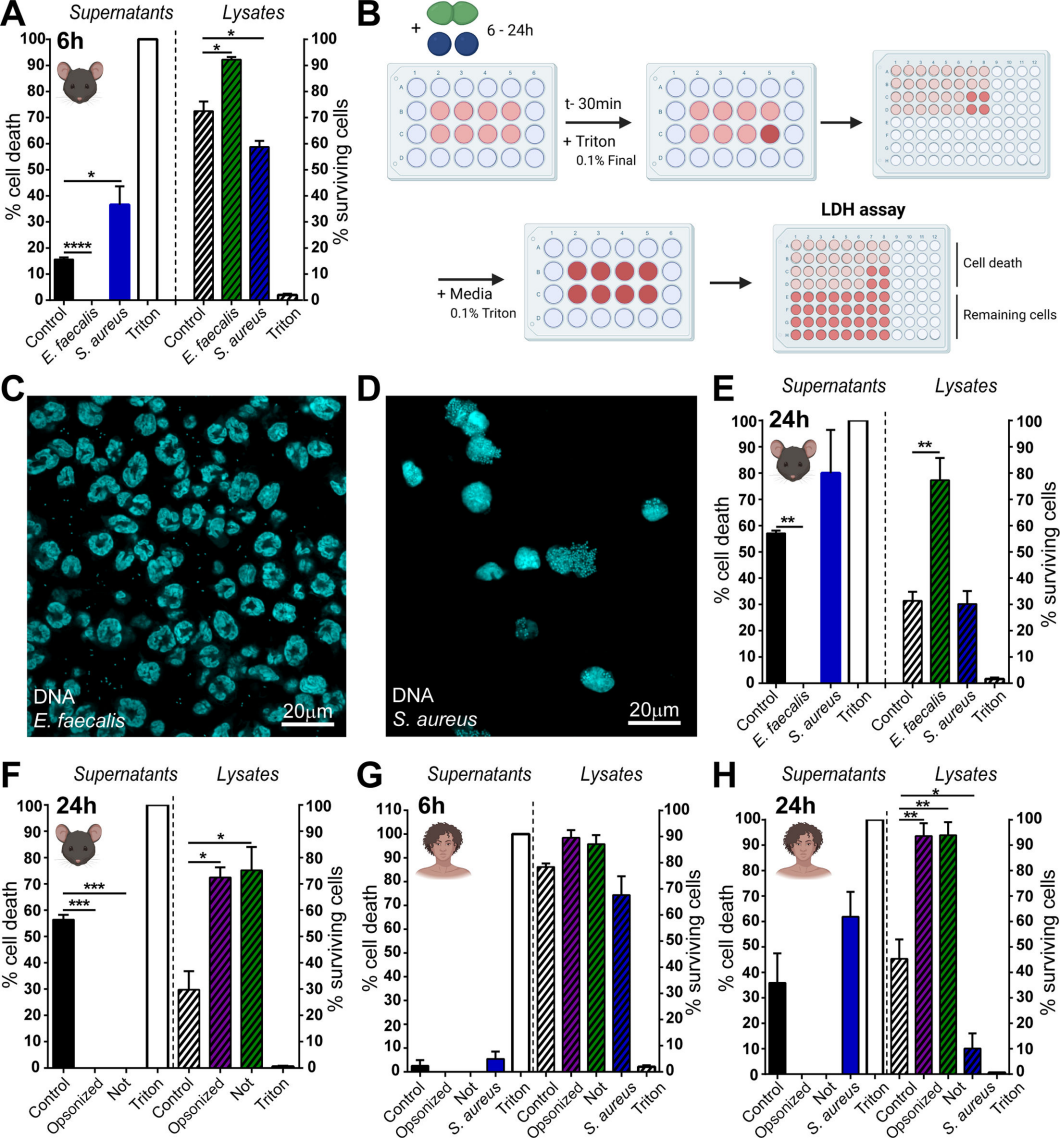
Suppression of spontaneous neutrophil cell death by *E. faecalis* infection. Neutrophils were infected with *E. faecalis* OG1RF or *S. aureus* USA300 (MOI 1) for 6 h, followed by gentamicin exclusion of extracellular bacteria. (**A, E–H**) At the indicated time point, LDH assay was performed both on supernatants and remaining cells lysed identically in media. Total percentage cell death in both cases was calculated by comparison to 100% kill well (“Triton”). (**B**) Schematic diagram depicting the modified LDH assay, which determines both percentage cell death and percentage surviving cells for the given time point. Light pink depicts cell culture media during standard assay; dark pink indicates media once Triton has been added to lyse all remaining cells. An experiment is performed on cells in a 24-well plate before media was taken and assay performed in a 96-well plate. (**A, C–F**) Depict experiments with primary murine neutrophils; (**G, H**) depict human neutrophils. For (**F–H**) *E. faecalis* were additionally incubated prior to infection for 15 min in either 10% mouse (**F**) or human (**G, H**) serum (“opsonized”) or else 10% FBS (“not”). (**C, D**) At 6 h p.i., cells were fixed with 4% PFA and stained with propidium iodide to mark DNA, then imaged at 63× on a confocal microscope. Data (**A, E–H**) depict mean + SEM from *n* = 6, 4, 4, 3, and 3 experiments, respectively. Data were analyzed using one-way ANOVA with Dunnett’s multiple comparisons test. * denotes *P* < 0.05, **P < 0.01, ***P < 0.001, ****P < 0.0001. (**C, D**) are representative of *n* = 4 experiments.

Given the presence of intracellular *E. faecalis* within neutrophils in the murine wound bed, along with our knowledge that *E. faecalis* persists long term in these wound beds (30), we hypothesized that the bacteria may evade neutrophil killing to persist intracellularly. To examine this, we first assessed the impact of *E. faecalis* exposure on neutrophil viability. Primary neutrophils were isolated from murine bone marrow and exposed to *E. faecalis* OG1RF or *Staphylococcus aureus* USA300 (multiplicity of infection [MOI] 1) for 6 h. Cell death was assessed via lactate dehydrogenase (LDH) release into the culture supernatant. As expected, *S. aureus* infection increased cell death, consistent with previous reports that phagocytosis accelerates neutrophil apoptosis (18, 34). Unexpectedly, *E. faecalis* infection resulted in negligible or even negative (reported here as zero) cell death (Fig. 2A), likely due to bacterial interference with LDH readings (35). To correct for this, we used a modified LDH assay that couples measurements from both the supernatant and the cell lysate, allowing estimation of the surviving cells based on retained LDH (Fig. 2B). This revealed ~90% survival of *E. faecalis*-infected neutrophils at 6 h p.i., significantly more than 70% for the uninfected control or ~60% following *S. aureus* infection (Fig. 2A). Extending the infection for 18–24 h did not increase cell death beyond that of uninfected control cells (Fig. S2A and B). Immunofluorescence microscopy supported these findings, showing that *E. faecalis*-infected neutrophils are still attached to the coverslip, with intact multi-lobulated nuclei and minimal morphological signs of activation (Fig. 2C). By contrast, *S. aureus* infected neutrophils exhibited extensive cell death, with nuclear condensation and cells destroyed or overrun with bacteria (Fig. 2D). Together, these data suggest that *E. faecalis* infection delays or inhibits neutrophil death typically triggered by infection (18).

To further characterize the fate of infected neutrophils, we added a high dose of gentamicin directly to the media at 6 h p.i. to kill extracellular bacteria, preventing re-infection and enabling monitoring of any ongoing intracellular infection (Fig. S2C). At 18 h and 24 h p.i., only 30–40% of uninfected control neutrophils remained viable, similar to the spontaneous apoptosis observed in the absence of antibiotic treatment, whereas ~90% and ~75% of *E. faecalis*-infected neutrophils survived at the same time points, respectively (Fig. 2E; Fig. S2D). Given the previous reports that serum opsonization enhances *E. faecalis* uptake and killing (24, 26, 27), we tested whether opsonization affected neutrophil survival. Neutrophils were infected with *E. faecalis* opsonized with either 10% homologous mouse serum or treated with 10% heat-inactivated fetal bovine serum (FBS) as a non-opsonized control. At 24 h p.i., ~70% of *E. faecalis*-infected neutrophils remained viable, regardless of opsonization, in contrast to ~30% in control cells (Fig. 2F). We next validated this observation in primary human neutrophils infected with *E. faecalis* in the presence or absence of 10% autologous serum. At 6 h p.i., cell death was comparable across all conditions, except for a slight drop in *S. aureus-*infected neutrophils (Fig. 2G). By 24 h p.i., ~90% of *E. faecalis*-infected neutrophils remained viable, regardless of opsonization, compared to ~40% of control cells and ~10% of *S. aureus*-infected cells (Fig. 2H). Thus, infection with *E. faecalis* suppresses the cell death of both primary murine and human neutrophils, independent of opsonization.

To determine if this phenomenon is strain-specific, we tested seven additional *E. faecalis* strains, including vancomycin-resistant strain V583 (36) and clinical wound isolates (30, 37). All but one wound isolate (Ef_22) suppressed neutrophil death, comparable to OG1RF (Fig. S2E). However, LDH readings varied among strains, with some (OG1RF, Ef_33, Ef_40, Ef_49) showing negative, artifactual readings, while others (Ef_22, Ef_23, Ef_46, V583) provided more accurate readings (Fig. S2F). Despite these differences, most *E. faecalis* strains broadly extended neutrophil life span beyond that of uninfected controls.

### *E. faecalis* is engulfed by neutrophils and persists at late time points

**Fig 3:**
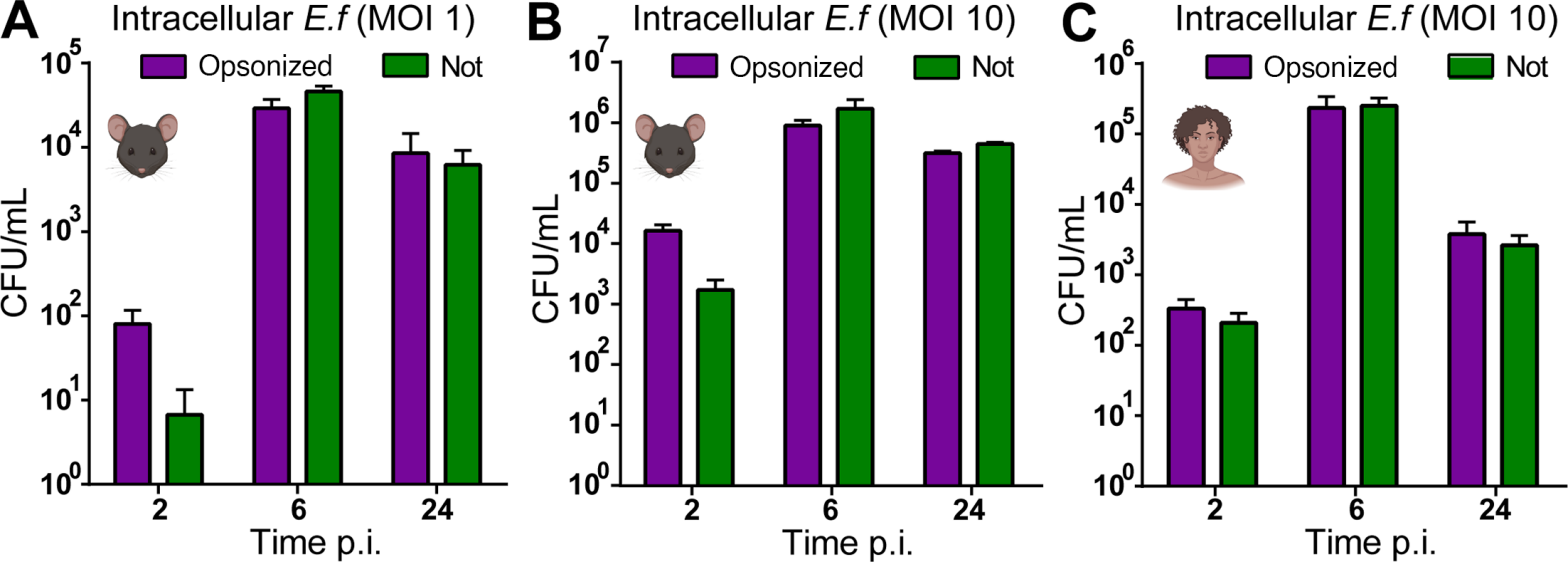
Intracellular persistence of phagocytosed *E. faecalis* in neutrophils to 24 h p.i. Murine (**A–B**) or human (**C**) neutrophils were infected with (**A**) MOI 1 or (**B, C**) MOI 10 *E. faecalis* OG1RF. The infection was halted at either 2 h (for 2 h time point) or 6 h, after which extracellular bacteria were killed by gentamicin exclusion. At the given time points, wells were gently washed once with PBS, then lysed in PBS + 0.1% Triton, and serially diluted on BHI for CFU enumeration. Data depict mean + SEM from *n* = 4, 4, and 4 independent experiments, respectively. For the non-opsonized infection condition, there is a significant difference between 2 h and 6 h (*P* = 0.0099) and 6 h and 24 h (*P* = 0.0112). For the opsonized infection condition, the *P* values for opsonized were close to significance (*P* = 0.0591, *P* = 0.0792 respectively). Data were analyzed by two-way ANOVA with Sidak’s multiple comparisons test.

To understand whether *E. faecalis*-mediated delay of neutrophil death also affects neutrophil function, we assessed bacterial uptake and intracellular persistence. Murine neutrophils were infected with MOI 1 (Fig. 3A) or MOI 10 (Fig. 3B), and CFU were enumerated from both the supernatant (extracellular) or the triton lysed neutrophils (intracellular) at 2, 6, and 24 h p.i. At 2 h p.i., opsonization enhanced infection numbers at 2 h p.i., but this advantage dissipated by 6 h p.i. (Fig. 3A and B). Surprisingly, intracellular *E. faecalis* persisted up to 24 h p.i., regardless of opsonization. Extracellular CFU increased until 6 h p.i., reflecting bacterial replication in the media. However, following gentamicin treatment at 6 h to kill extracellular bacteria, no viable extracellular CFU were detected at 24 h (Fig. S3A). Thus, *E. faecalis* are readily engulfed by murine neutrophils and can persist intracellularly at late time points.

In human neutrophils infected with MOI 1, intracellular CFU were undetectable at 2 h p.i. and rose to ~10^3^ at 6 h p.i., regardless of opsonization. By 24 h p.i., only low levels of opsonized intracellular *E. faecalis* were detectable (Fig. S3B). By contrast, infection with MOI 10 resulted in detectable intracellular CFU at all time points (Fig. 3C). The levels of *E. faecalis* (MOI 1) in murine neutrophils (Fig. 3A) were comparable to those in human neutrophils (MOI 10) (Fig. 3C), suggesting that human neutrophils are more effective at clearing intracellular *E. faecalis*. At 24 h p.i., ~30% of infected human neutrophils (MOI 10) reported cell death (Fig. S3C). Thus, *E. faecalis* can persist within viable human neutrophils for up to 24 h.

### *E. faecalis* resides within membrane-bound vacuoles in neutrophils

**Fig 4:**
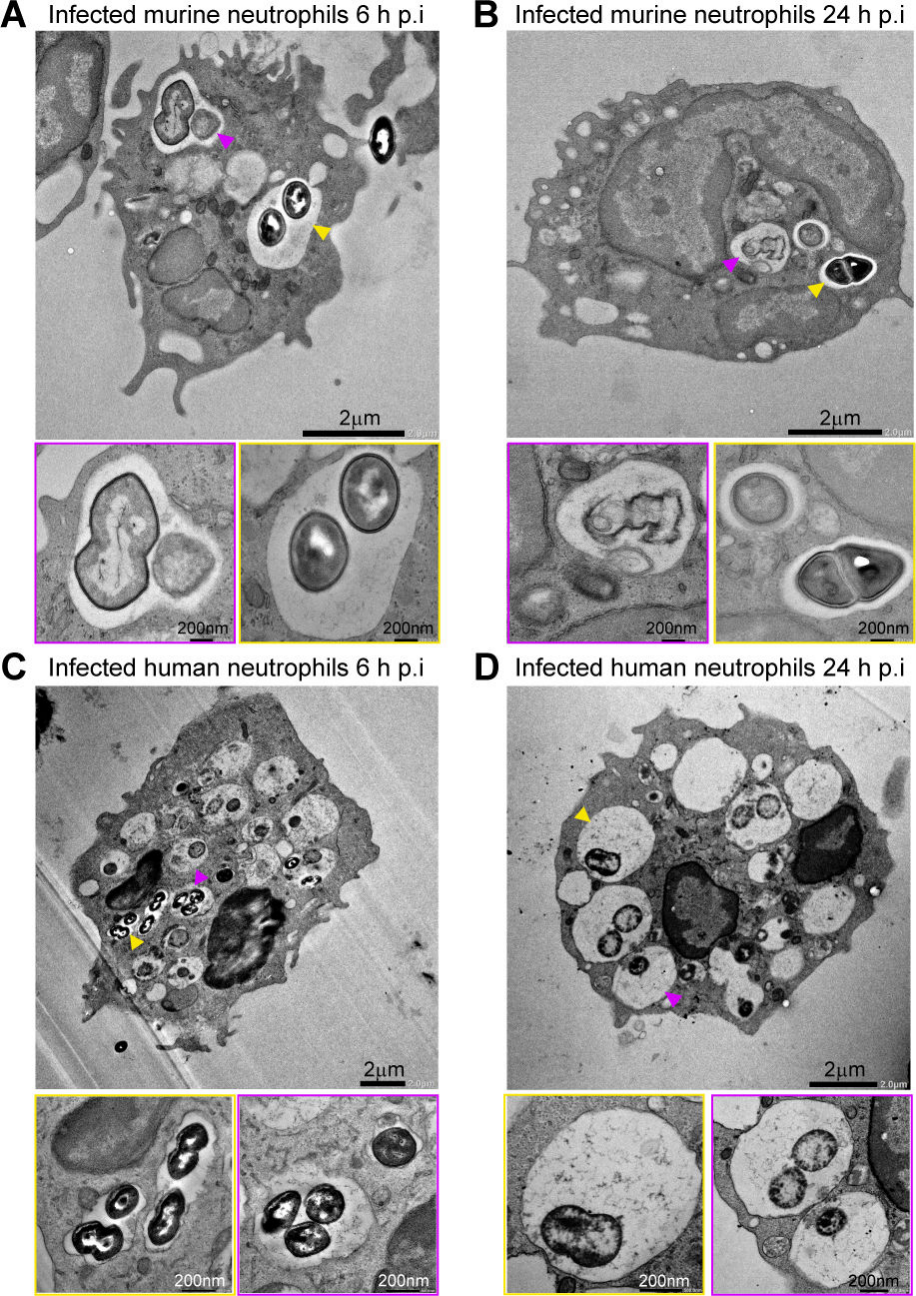
Localization of *E. faecalis* within membrane-bound vacuoles in murine and human neutrophils. (**A–B**) Murine or (**C–D**) human neutrophils were infected with (**A–B**) MOI 1 or (**C–D**) MOI 10 of *E. faecalis* OG1RF for 6 h, followed by gentamicin exclusion of extracellular bacteria. At (**A, C**) 6 h or (**B, D**) 24 h p.i., cells were washed with PBS and then fixed with 2.5% glutaraldehyde. Cells were imaged via transmission electron microscopy. Depict individual cells from their respective time points, representative of at least six cells per condition. Yellow arrowheads mark larger, intact membrane-bound compartments, with magenta arrowheads indicating vacuoles with partial or degraded membranes.

To gain insight into the intracellular niche for *E. faecalis* within neutrophils, we again infected murine and human neutrophils with *E. faecalis* OG1RF at MOI 1 or MOI 10, respectively, for 6 h, followed by transmission electron microscopy (TEM) (Fig. 4). At 6 h p.i., infected neutrophils retained their multi-lobular nuclei and intact plasma membrane, with pseudopodia extending from the cell surface (Fig. 4A and C; Fig. S4A). Distinct subcellular structures were visible, including specific (lighter/less electron dense) and azurophilic (darker/more electron dense) granules, glycogen granules, and strands of endoplasmic reticulum (ER). Internalized *E. faecalis* were consistently observed, frequently in large, spacious membrane-bound vacuoles (yellow arrowheads), which were more numerous within human neutrophils (likely reflecting the higher MOI used). In some instances, disrupted vacuolar membranes and visible damaged or degraded bacteria were also observed (magenta arrowheads). Multiple, separate compartments containing one or more bacteria were detected; however, they may represent segments of a single larger compartment. At 24 h p.i. (Fig. 4B and D; Fig. S4B), infected neutrophils continued to maintain their overall cell morphology, including the nuclear membrane, granule content, and intact ER. In human neutrophils, numerous large, empty vacuoles were evident (Fig. 4C), potentially reflecting cleared phagosomes or early apoptotic features. Similarly, several small vacuoles near the plasma membrane were visible in murine neutrophils (Fig. 4B). Internalized bacteria in a combination of membrane-bound and membrane-disrupted compartments were visible in both murine and human neutrophils. Notably, *E. faecalis* within intact membrane-bound vacuoles appeared morphologically healthy, while those exposed to the cytoplasm or associated with granules showed signs of degradation and disintegration. By contrast, control uninfected neutrophils at 24 h p.i. exhibited widespread vacuolization, nuclear breakdown, and cellular degradation (Fig. S4C and D). Uninfected cells that could be visualized at all were overrun with vacuoles and degrading components, with breakdown of the nuclear membrane and nucleus. These striking differences provide ultrastructural confirmation of both the extended neutrophil survival observed in LDH assays (Fig. 2C and D) and support the intracellular persistence of *E. faecalis* within both murine and human neutrophils (Fig. 3A and B).

### *E. faecalis* replicates intracellularly within neutrophils

**Fig 5:**
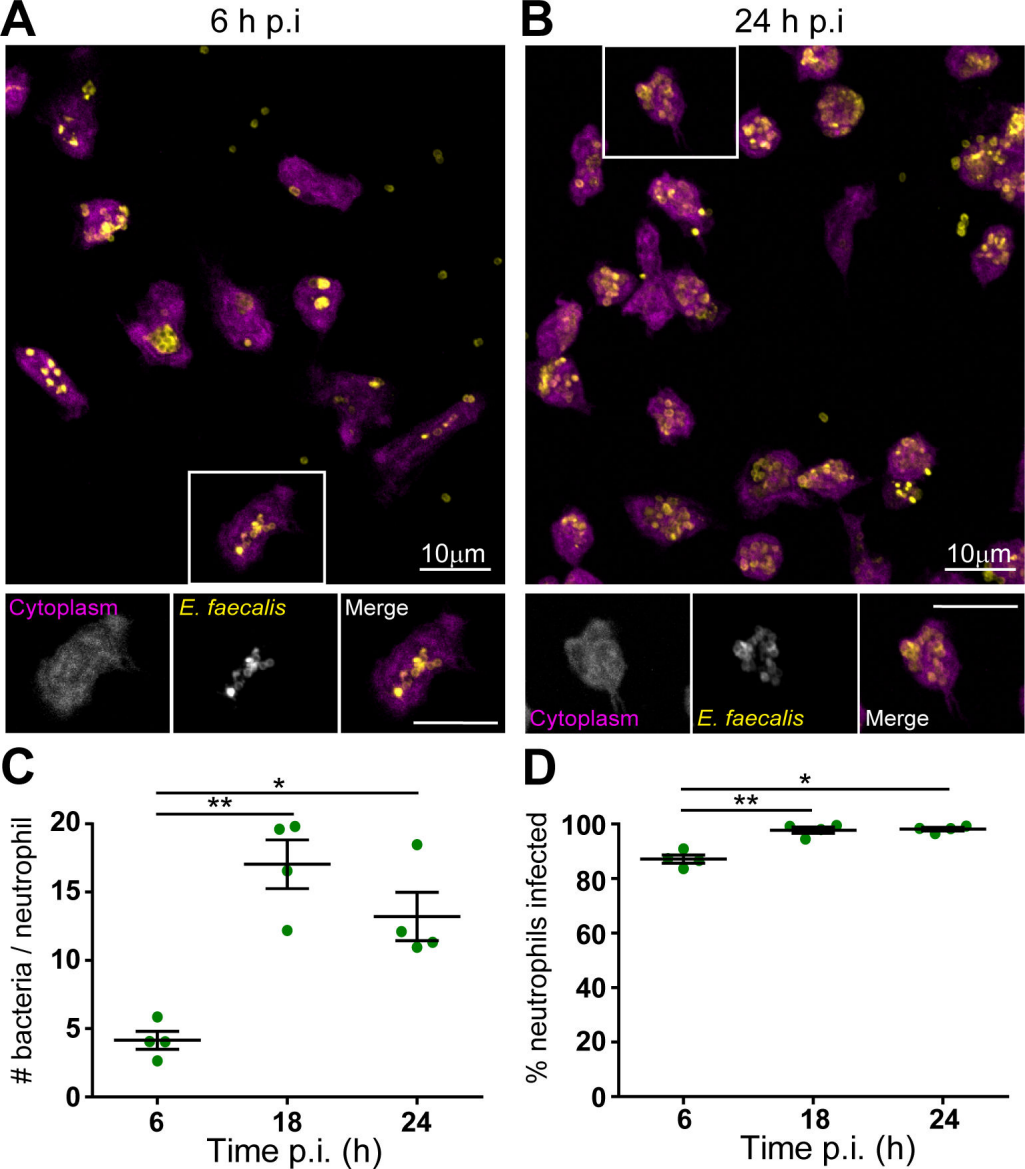
Engulfment and intracellular persistence of *E. faecalis* within murine neutrophils to 24 h p.i. Murine neutrophils were pre-stained with CellTracker Blue (cytoplasm, magenta), then infected with *E. faecalis* OG1RF for 6 h, followed by gentamicin exclusion of extracellular bacteria. At (**A, C**) 6 or (**B, D**) 24 h p.i., cells were fixed with 4% PFA and stained for Streptococcal Group D antigen (*E. faecalis* bacteria, yellow) before being imaged at 63× on a confocal microscope. (**C–D**) Images from (four per condition, minimum 50 cells total) were blindly quantified to determine the total number of bacteria, neutrophils, and infected neutrophils. (**A–B**) Depict a single experiment, representative of *n* = 4 independent experiments. (**C–D**) Depict mean ± SEM, from the same *n* = 4 independent experiments. Data were analyzed by one-way ANOVA with Tukey’s multiple comparisons test; * denotes P < 0.05, **P < 0.01.

To investigate whether *E. faecalis* replicates intracellularly within neutrophils, we stained murine neutrophils with the cytoplasmic dye CellTracker Blue, before infection with MOI 1 of *E. faecalis* OG1RF for 6 h. Intracellular bacteria were visible at 6 h p.i. (Fig. 5A) and 24 h p.i. (Fig. 5B). Blinded quantification of intracellular bacteria per neutrophil revealed a 3-fold increase between 6 and 18 h p.i., which persisted to 24 h p.i. (Fig. 5C). We also observed a modest but significant increase in the percentage of infected neutrophils between 6 h and later time points (Fig. 5D). Since extracellular bacteria were killed by gentamicin from 6 h p.i., this suggests that the ~25% cell death observed at 24 h via the LDH assay (Fig. 2D) predominately comprised uninfected neutrophils.

**Fig 6:**
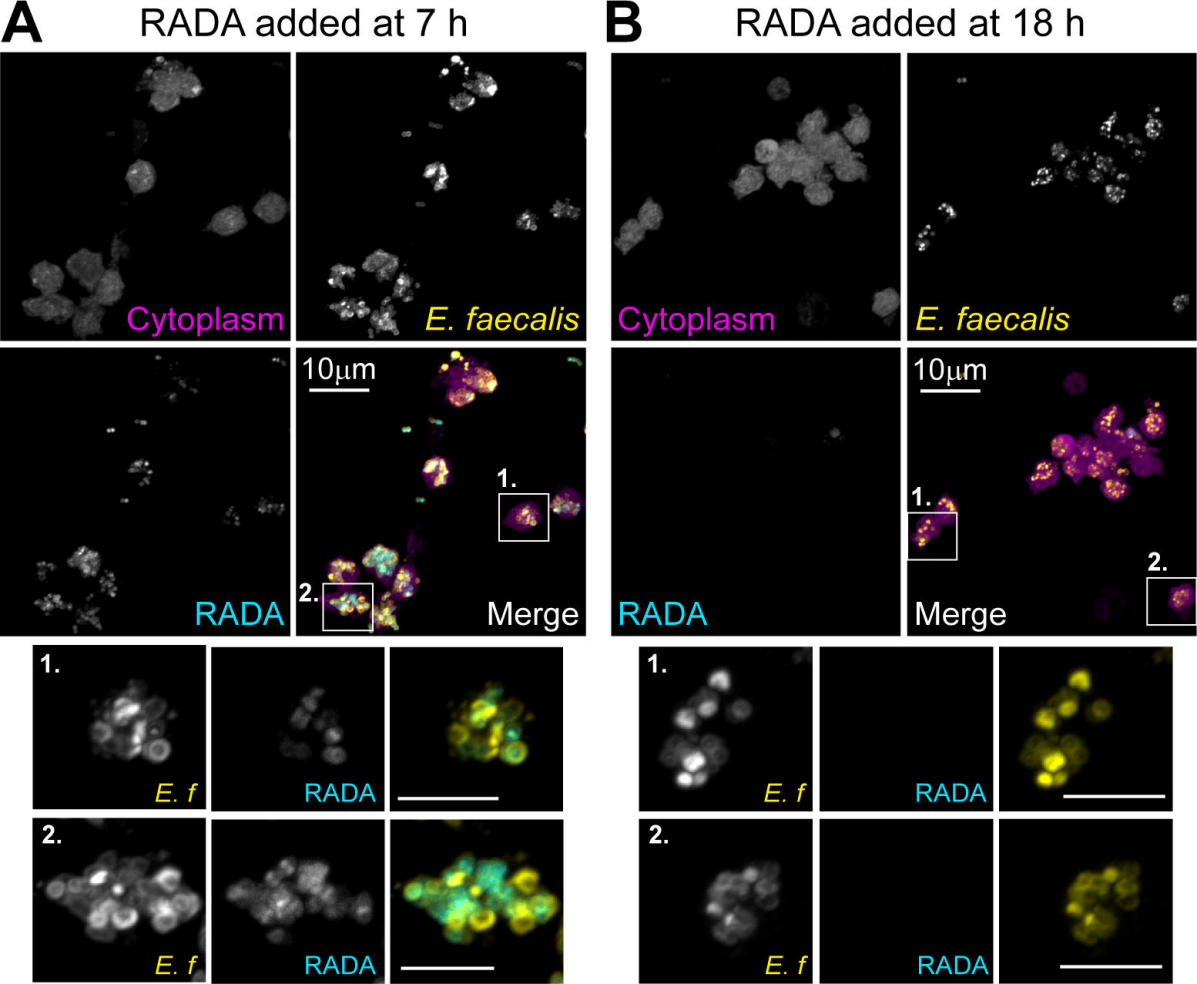
Intracellular replication of *E. faecalis* within murine neutrophils, between 6 and 18 h p.i. Murine neutrophils were pre-stained with CellTracker Blue (cytoplasm, magenta), then infected with *E. faecalis* OG1RF for 6 h, followed by gentamicin exclusion of extracellular bacteria. At (**A**) 7 h or (**B**) 18 h p.i., RADA (fluorescent D-amino acid incorporated into bacterial cell walls during synthesis, cyan) was added into the high-dose gentamicin media. At 24 h p.i., cells were fixed with PFA and stained for Streptococcal Group D antigen (*E. faecalis* bacteria, yellow) before being imaged at 63× on a confocal microscope. Scale bar in insets depicts 10 mm. Images depict a single experiment, representative of *n* = 4 independent experiments.

To further confirm *E. faecalis* replication within neutrophils, we used the fluorescent D-amino acid probe RADA, which incorporates into newly synthesized bacterial peptidoglycan (38). Infected murine neutrophils were treated with gentamicin at 6 h p.i., followed by RADA addition at either 7 or 18 h p.i. (i.e., 1 or 12 h after gentamicin addition), and imaged at 24 h p.i. When RADA was added at 7 h p.i., we observed fluorescence incorporation in the cell wall of intracellular *E. faecalis*, indicating active replication (Fig. 6A). By contrast, RADA incorporation was minimal when added at 18 h p.i. (Fig. 6B), suggesting that *E. faecalis* replicate intracellularly within murine neutrophils between 6 and 18 h, followed by a persistent non-replicative phase from 18 h onward.

### Infected neutrophils are capable of pro-inflammatory cytokine signaling

**Fig 7:**
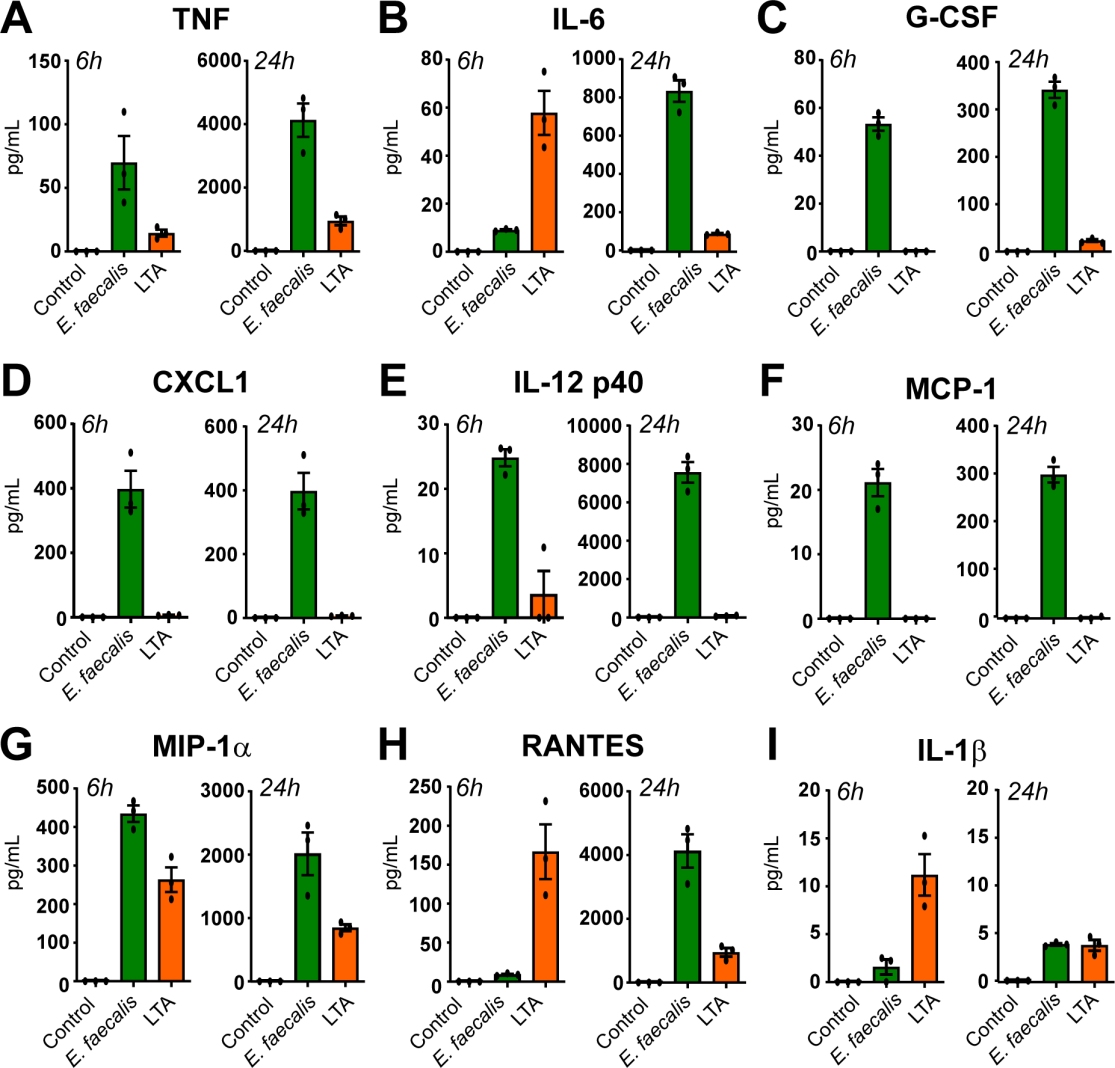
Robust neutrophil cytokine response induced by *E. faecalis* infection. Murine neutrophils were infected with MOI 1 of *E. faecalis* OG1RF or stimulated with 10 ng/mL lipoteichoic acid (LTA) for 6 h, followed by gentamicin exclusion of extracellular bacteria. At either 6 or 24 h p.i., supernatants were removed and assessed via multiplex cytokine analysis. Cytokines assessed were (**A**) TNF, (**B**) IL-6, (**C**) C-CSF, (**D**) CXCL1, (**E**) IL-12 p40, (**F**), MCP-1, (**G**) MIP-1α (**H**) RANTES and (**I**) IL-1β. Data depict mean ± SEMfrom three independent experiments.

Finally, we sought to determine whether the prolonged intracellular occupation and replication of *E. faecalis* within neutrophils was immunologically “silent” or accompanied by cytokine signaling. Murine neutrophils were infected, left uninfected, or treated with 10 µg/mL lipoteichoic acid (LTA) as a positive stimulus control. Supernatants were collected at 6 and 24 h p.i. and assessed for pro-inflammatory cytokine release (Fig. 7). At 6 h p.i., *E. faecalis*-infected neutrophils secreted modest levels of tumor necrosis factor (TNF), granulocyte colony-stimulating factor (G-CSF), chemokine CXCL1, and monocyte chemoattractant protein-1 (MCP-1). High levels of macrophage inflammatory protein MIP-1α were also detected at levels similar to LTA-treated controls. By contrast, secretion of interleukin 6 (IL-6), chemokine RANTES/CCL5, and IL-1β was induced by LTA, but not by *E. faecalis* at this early time point. By 24 h p.i., cytokine production by *E. faecalis*-infected neutrophils was markedly increased. High levels of TNF, IL-6, G-CSF, CXCL1, IL-12 p40, MCP-1, MIP-1α, and RANTES were detected (Fig. 7A through H). High levels of MIP-1β were also detected at this time, exceeding the limit of accurate quantification by the assay (data not shown). However, in agreement with our data reporting the suppression of neutrophil death by *E. faecalis* infection (Fig. 2), minimal IL-1β was detected (Fig. 7I). Thus, *E. faecalis-*infected neutrophils have the capacity to signal for immune cell recruitment and activation, in particular other innate immune cells such as macrophages and additional neutrophils.

## DISCUSSION

Neutrophils are highly reactive, inherently short-lived immune cells. While examples of bacterial pathogens persisting and/or replicating with macrophages are numerous (39), similar examples in neutrophils are exceedingly rare. Indeed, phagocytosis of most bacteria accelerates neutrophil death (40), as reported for *Escherichia coli* (15), *Streptococcus pneumoniae*, *Streptococcus pyogenes*, *Mycobacterium tuberculosis*, *Listeria monocytogenes*, and *Borrelia hermsii* (41). Some bacteria, such as *S. aureus*, induce rapid neutrophil death via necrosis or necroptosis (42).

Only a handful of intracellular pathogens—*Chlamydia pneumoniae* (20), *Neisseria gonorrhoeae* (43), *Francisella tularensis* (44), *Leishmania major* (45), and *Anaplasma phagocytophilum* (46)—have been reported to delay neutrophil death or exploit neutrophils as a niche for replication. Thus, the finding that *E. faecalis*, a traditionally extracellular, biofilm-associated pathogen, can suppress neutrophil death and persist and replicate inside these immune cells was highly unexpected. This discovery reshapes our understanding of neutrophil function in response to *E. faecalis* and helps resolve the long-standing disconnect between *in vitro* studies reporting efficient neutrophil killing and *in vivo* observations of persistent infection despite an acute immune response (30). Previous research on *E. faecalis*-neutrophil interactions demonstrated bacterial adherence (23), phagocytosis (24), and killing (26–28), but was limited to very early time points (≤2 h p.i.). This narrow temporal approach to studying neutrophil-bacterial interactions is commonplace, possibly leading to underestimation of bacterial capacities to resist neutrophil killing. Similarly, phagocytosis has often been taken as the endpoint, without verifying subsequent bacterial killing. For example, research with extraintestinal pathogenic *E. coli* (ExPEC) revealed that most intracellular bacteria remained viable 3.5 h p.i. following phagocytosis by neutrophils (47). Similarly, neutrophils obtained from the peritoneal cavity of *S. aureus-*infected mice contained viable bacteria capable of establishing infection when transferred into naïve animals (48).

This work expands our evolving understanding of *E. faecalis* beyond being an extracellular pathogen (13). Indeed, increasing evidence shows that *E. faecalis* can be taken up by and persist within a range of mammalian cells including endothelial cells (49), epithelial cells (50, 51), and macrophages (11, 52, 53). Recent studies have shown that *E. faecalis* not only persists but also replicates intracellularly within keratinocytes, RAW 264.7 macrophages, and hepatocytes using BrdU or RADA staining following extracellular antibiotic killing (12, 54). Interestingly, intracellular bacteria isolated from infected keratinocytes were internalized by naïve keratinocytes at a nearly 10-fold higher rate (12), similar to observations of *S. pyogenes* uptake and subsequent reinfection of macrophages (55). Extension of this work would logically involve examining the level of neutrophil cell death suppression at later time points beyond 24 h and determining the viability and re-infection capacity of the intracellular *E. faecalis*.

*In* vivo, neutrophils accumulate in large numbers in both control and *E. faecalis* acutely infected wounds; however, neutrophil numbers decline in uninfected wounds as healing progresses but remain elevated in *E. faecalis*-infected wounds, where healing is delayed (30). In the current study, *E. faecalis*-infected neutrophils were fully capable of pro-inflammatory signaling, with many of these potent recruiters and activators of immune cells. For example, TNF and IL-6 can activate endothelium and promote inflammation; G-CSF mobilizes and stimulates neutrophil production; CXCL1 (KC) and MIP-1α/β attract additional neutrophils and monocytes; MCP-1 is a key chemoattractant for monocytes/macrophages; and IL-12 can drive Th1-type immune responses. In a wound setting, this cocktail of cytokines would likely amplify the immune response, recruiting waves of leukocytes (possibly also increasing the number of intracellular niches) to the site of infection, as well as driving enhanced activation and antimicrobial capacity of existing macrophages. This is in keeping with previous *in vivo* wound infection findings at 24 h p.i. (30); however, here IL-1 β was also found to be upregulated (unlike in our *in vitro* infected neutrophils), suggesting that the source of IL-1β in the wound is coming from elsewhere, or that the more complex *in vivo* environment is potentiating a different cytokine profile from the neutrophils. Prolonged inflammation can impart tissue damage and delayed healing; thus, it would be pertinent to also examine cytokine responses from *E. faecalis*-infected neutrophils at later time points and to confirm whether these match the drop in pro-inflammatory cytokine production seen in murine wounds at 72 and 96 h p.i. (56).

By prolonging neutrophil life span, *E. faecalis* may create a temporary intracellular niche that supports its persistence and replication, leading to increased bacterial burden and enhanced inflammation at the wound site. It is likely that a feedback loop exists involving ongoing phagocyte recruitment, phagocytosis, and intracellular persistence, with short-lived neutrophils facilitating subsequent longer-term persistence in macrophages (11, 12). A similar mechanism has been described for *Leishmania major*, an intracellular parasite that exploits neutrophils as a “Trojan horse” to gain entry into host macrophages (45). *Leishmania* delays neutrophil apoptosis while inducing MIP-1β secretion, enhancing macrophage engulfment of infected neutrophils, within which the internalized bacteria survive and replicate (45). This process was visualized *in vivo* in a murine model in which dermal macrophages acquired parasites via direct transfer from, or efferocytosis of, infected neutrophils (57). Future work should clarify the relative contributions of extended neutrophil life span versus ongoing neutrophil recruitment during *E. faecalis* wound infection.

Our study did not directly assess the contribution of intracellular persistence to biofilm-associated wound infection, in part because it is difficult to differentiate between persistence and reinfection. Ongoing work seeks *E. faecalis* mutants that cannot replicate in neutrophils to more definitively answer this question. Nonetheless, the ability of *E. faecalis* to survive and replicate within neutrophils could provide a transient refuge from antibiotics and immune attack, functioning as a “Trojan horse” mechanism, whereby viable intracellular bacteria are later released upon neutrophil death or uptake by macrophages to reseed infection or initiate new biofilm foci. We therefore propose that intracellular persistence may represent an underappreciated facet of *E. faecalis* biofilm-associated chronic wound infection, a hypothesis to be tested in future *in vivo* work.

TEM of infected neutrophils confirmed that, despite infection, murine and human neutrophils retained their morphology and multilobulated nuclei, in striking contrast to uninfected control neutrophils. TEM also revealed that *E. faecalis* reside within spacious, membrane-bound vacuolar compartments at 6 and 24 h p.i. This observation is consistent with previous ultrastructural studies of human neutrophils infected with closely related *Enterococcus faecium*, which similarly showed bacteria within discrete, membrane-bound compartments, typically surrounded by substantial “empty” space (27). TEM of ExPEC within neutrophils also revealed the occasional presence of “spacious phagosomes” alongside “tight phagosomes” (47), which are more commonly observed (58). Interestingly, TEM of neutrophils collected from murine peritoneal lavages 24 h p.i. with a *S. aureus* clinical blood isolate also revealed bacteria within “spacious phagosomes,” while a mutant lacking the virulence regulator Sar was predominately within “tight phagosomes” (48).

The mechanism by which *E. faecalis* delays neutrophil death and resists antimicrobial clearance to permit persistence and replication remains to be determined. It is well documented that purified LTA (59) and LPS (60) can delay spontaneous neutrophil death, in the case of LTA via Toll-like receptor 2 and pattern recognition receptor CD14 (59). Similarly, growth factors, such as G-CSF (60, 61) and GM-CSF (62, 63), also prolong neutrophil life span, largely through signaling pathways that converge on Nf-κB activation (64). Thus, in the context of *E. faecalis* infection, one possibility is that initial recognition of LTA on phagocytosed *E. faecalis*, followed by robust production of G-CSF by infected neutrophils, promotes neutrophil survival via NF-kB pathways. However, G-CSF signaling can also enhance neutrophil phagocytic and antibacterial activity (65), which is not consistent with the observed persistence of intracellular bacteria in our study. Further, pro-inflammatory cytokines, such as TNF (66), typically drive neutrophils toward apoptosis, although conflicting reports suggest the impact of this cytokine on neutrophil survival may be time-dependent (67). Adding further complexity, *E. faecalis* can suppress NF-κB signaling in macrophages (68), raising the possibility that similar immune evasion tactics could be active in neutrophils.

Extensive work has been carried out to characterize the mechanisms by which *F. tularensis* suppresses neutrophil function to establish colonization (22, 44, 69, 70). *F. tularensis* inhibits the generation of reactive oxygen species (ROS) during neutrophil infection, even in the presence of compounds that stimulate ROS production (44, 69). *F. tularensis* infection also interferes with apoptotic pathways, blocking the cleavage and activation of caspase-3, caspase-8, and caspase-9 (70). Finally, *F. tularensis* preserves neutrophil mitochondrial integrity by preventing Bax translocation and Bid processing during infection (22). These host pathways offer a useful framework for identifying the molecular mechanisms by which *E. faecalis* may similarly subvert neutrophil death and promote intracellular persistence. Detailed characterization of intracellular ROS levels or impact on apoptotic pathways during *E. faecalis* infection is lacking; however, in murine neutrophils, ROS generation has been associated with bacterial killing as early as 4 h p.i. (71).

The deliberate manipulation of immune cell death pathways is a well-established therapeutic approach in oncology (72), and there is growing interest in applying similar approaches to infectious disease (73, 74). Understanding how *E. faecalis* suppresses neutrophil apoptosis may therefore uncover novel therapeutic targets. Such insights are particularly relevant for tackling chronic wound infection, as well as a broader array of *E. faecalis-*driven pathologies, including infective endocarditis and urinary tract infection (1, 2, 68, 75).

## MATERIALS AND METHODS

### Bacterial strains and culture

All bacterial strains, including *Enterococcus faecalis* strain OG1RF (76), *E. faecalis* strain OG1RF pDasher (33), *E. faecalis* strain VR583 (36), and *Staphylococcus aureus* strain USA300LAC (77), were all grown on brain heart infusion (BHI) medium (BD Biosciences) agar (BD Biosciences). Clinical wound swab isolates were all obtained from Tan Tock Seng Hospital, Singapore (30, 37). For overnight cultures, a single colony from a fresh bacterial streak was inoculated into a 10 mL static culture and incubated overnight at 37°C. After two washes with PBS, *E. faecalis* was adjusted to OD_600_ nm = 0.5 (~3 × 10^8^ CFU/mL) and *S. aureus* OD_600_ nm = 1.5 (5 × 10^8^ CFU/mL) to enable MOI calculations (and serially diluted on BHI agar to subsequently confirm).

### Murine model of wound infection

Mouse wound infections were performed as previously described (30). Briefly, 6- to 9-week-old mice were anesthetized using isoflurane. All hair was then removed from the backs of mice via (electric) shaver followed by hair removal cream (Nair). Following 70% ethanol disinfection, a wound was made using a 6 mm sterile biopsy punch (Integra Lifesciences). Ten microliters of bacterial inoculum (10^6^ CFU) were added to the wound, after which Tegaderm adhesive dressing (3M) was applied. Twenty-four hours post-infection, a ~1 cm^2^ square piece of tissue (encompassing the wound) was collected and processed based on downstream experimentation.

For cryo-sectioning, tissues were excised with approximate dimensions of 1.5 cm × 1.5 cm and placed on Whatman filter paper (Sigma-Aldrich). Tissues were immediately submerged in 2 mL of 4% paraformaldehyde and stored in 4℃ for 8 h. Following that, tissues were submerged in 15% and 30% sucrose for 24 h each. Fixed tissues were trimmed and placed in OCT (Sakura Finetek)-filled cryomolds (Sakura Finetek) and flash-frozen before long-term storage at −80°C.

For flow cytometry, wounds were collected in 500 mL of serum-free media, to which Liberase (Merck) was added at a final concentration of 200 mg/mL, and the samples were placed at 37°C for 1 h with occasional agitation to dissociate host cells. An equal volume of FACS staining buffer (FCSB; PBS plus 2% heat-inactivated fetal bovine serum [FBS, Gibco] and 0.2 mM EDTA [Gibco]) was then added to stop the reaction. Cells were strained using a 40 µm cell strainer (SPL Life Sciences), centrifuged, and resuspended in 100 mL FCSB. Following counting on a Countess 3 cell counter (Thermo Fisher), ~5 × 10^6^ cells were taken for further processing and examination via flow cytometry.

### Murine neutrophil extraction and culture

Murine bone marrow neutrophils were obtained as previously described (71). Briefly, femurs and tibias were removed from 6- to 9-week-old female C57BL/6 mice, placed on ice, and flushed with cold human plasma-like media (HPLM, Gibco) using a 25G needle (Terumo). Cells were resuspended in MACs buffer (PBS [Gibco] plus 0.5% FBS, 2 mM EDTA) and run through a 40 µm cell strainer before being isolated via negative selection using a Mojosort mouse neutrophil isolation kit (Biolegend). Cells were first incubated for 15 min on ice with a biotin-labeled antibody cocktail, followed by incubation with streptavidin-conjugated magnetic beads for another 15 min on ice. The washed resuspension was then added to an LS column (Miltenyi Biotec) and placed on a magnetic separator (Miltenyi Biotech). The flow-through, containing purified neutrophils, was collected ( ~6–8 M/mouse). For the majority of subsequent experimentation, cells were resuspended in HPLM plus 10% heat-inactivated FBS and plated into 24-well plates that had been precoated with 0.0001% poly-L-lysine (Sigma-Aldrich) at 500,000 cells per well in 1 mL of medium. For immunofluorescence, cells were first resuspended in Opti-Mem (Gibco), plated onto 12 mm glass coverslips (Fisher Scientific) (also precoated with poly-L-lysine), and stained with cytoplasmic dye CellTracker Blue (Thermo Fisher) at a final concentration of 5 mM for 30 min, before the media was removed and replaced with fresh HPLM.

### Human neutrophil extraction and culture

Primary human neutrophils were obtained from human whole blood using negative selection with the MACSxpress Whole Blood Neutrophil Isolation Kit (Miltenyi Biotec), following the manufacturer’s instructions. Briefly, 8 mL of whole blood (freshly collected into EDTA-coated tubes at Fullerton Health, NTU) was combined with an antibody cocktail and buffer solution. After thorough mixing, this was placed on a strong magnet (Miltenyi Biotec) and left for 15 min. All antibody- and magnetic bead-coated cells then coagulate, leaving residual supernatant containing neutrophils alone, which was removed and resuspended in PBS for counting. The yield was ~16-24 M cells. Neutrophil purity was determined using antibodies against human CD45 and CD15 (2D1, HI98, Thermo Fisher).

### Immunofluorescence microscopy

For *in vitro* neutrophils on coverslips, at the given time points, 750 mL of media was removed from each well and replaced with 250 mL 8% PFA (final concentration 4%) and fixed for 15 min at 37°C. After three washes with PBS, cells were permeabilized with 0.1% Triton for 5 min, then blocked for 1 h with 1% bovine serum albumin (BSA, Sigma-Aldrich). For cryosections on glass slides, sections were first incubated in IHC Antigen Retrieval Solution (Invitrogen) for 20 min at 60°C, before being washed thrice with Milli-Q (Elgar Labwater) and once with PBS. Containing cells were permeabilized by dipping into 0.5% Triton in PBS for 5 min, followed by three washes in PBS. Sections were then blocked using blocking serum (PBS plus 5% FBS, 5% BSA, 0.05% Triton) for 1 h. Both were then incubated overnight at 4 °C with polyclonal rabbit antibodies against Streptococcus Group D antigen (1:500 dilution from whole serum aliquot) (#12-6231D, American Research Products) in either 1% BSA (coverslips) or antibody diluent solution (PBS plus 1% FBS, 1% BSA, 0.05% Triton). After three washes with PBS, cells were then incubated with goat anti-rabbit Alexa-488 1:1000 (A11034, Invitrogen), in the same diluent for 1 h at RT. Samples were washed three times with PBS, and DNA was then stained with 400 µM propidium iodide (Invitrogen) (coverslips) for 15 min, or with 10 µg/mL DAPI (Thermo Fisher) for 5 min. Samples were then mounted using ProLong Glass Antifade Mountant (Thermo Fisher) and imaged at 63× magnification on a Carl Zeiss LSM 780 laser scanning confocal microscope (NTU Optical Bio-Imaging Center [NOBIC] imaging facility, SCELSE).

### Flow cytometry

Flow cytometry was performed as described previously (12). Briefly, excised skin samples were placed in 1.5 mL of liberase (2.5 U/mL) prepared in cell culture media. The mixture was incubated with agitation for 1 h at 37°C with 5% CO_2_. Dissociated cells were then passed through a 70 mm cell strainer and spun down at 1,350 rpm for 5 min at 4°C. The enzymatic solution was then aspirated, and cells were blocked in 500 µL of fluorescence-activated cell sorting (FACS) buffer (2% FBS and 0.2 mM EDTA in PBS [Gibco; Thermo Fisher Scientific]). Cells (˜10^7^ per sample) were then incubated with 10 µL of Fc-blocker (anti-CD16/CD32 antibody; BioLegend) for 30 min, followed by incubation with either an anti-mouse CD45, CD11b, and Ly6G (for neutrophils) or CD45, CD11b, and F4/80 (for macrophages) (all from BioLegend, 1:100 dilution) for 30 min. Cells were again washed in FACS buffer before being fixed in 4% PFA for 15 min at 4°C. Cells were washed a final time, then resuspended and analyzed using the BD LSRFortessa X-20 Cell Analyzer (Becton Dickinson). Compensation was performed using the AbC Total Antibody Compensation Bead Kit (Thermo Fisher Scientific) according to the manufacturer’s instructions.

### Lactate dehydrogenase (LDH) assay

Neutrophil death was determined using a cytotoxicity detection kit (Merck), following the manufacturer’s instructions, with modifications to determine both the approximate cell death from cell supernatants and the remaining cell lysates to overcome potential bacterial interference with LDH readings (35). Briefly, alongside experimental condition wells, an identical well of cells was lysed with the addition of 10% Triton (final concentration 0.1%) (Sigma-Aldrich) 30 min prior to the experimental endpoint. This well was then flushed and the supernatant used as the “100% Triton kill” well to which all other cell death is determined in proportion to. Following initial sampling of supernatants, all media was removed from each well and replaced with 1 mL of HPLM + 0.1% Triton for 15 min (i.e., to lyse all remaining viable cells). This was then sampled and run alongside initial supernatant samples. After centrifugation, 50 µL of sample was combined with 50 mL of freshly prepared reagent and left for 30 min, after which the plate was read at 490 nm in a Tecan Infinite 200 PRO spectrophotometer. Note that for 24 h assay readings, gentamicin was added directly to the well (final concentration 200 µg/mL) at 6 h p.i.

### Bacterial clearance assay

Following OD normalization, *E. faecalis* bacteria were incubated with 10% mouse serum (opsonized) or 10% heat-inactivated FBS (non-opsonized) for 15 min at 37°C, after which they were washed and resuspended in PBS and used for infection. Neutrophils were infected with MOI 1 or MOI 10 of bacteria for 2 or 6 h, after which the supernatants were sampled (and serially diluted on BHI agar for CFU enumeration of extracellular bacteria). All infectious media was then removed and replaced with 1 mL of HPLM containing 200 µg/mL gentamicin for 30 min. The media was then removed, and neutrophils were washed carefully twice with PBS before being lysed with 1 mL PBS containing 0.1% Triton. This solution was then serially diluted as described above for CFU enumeration of intracellular bacteria. For the 24 h time point, at 6 h, the media was removed and replaced with HPLM containing 200 mg/mL gentamicin for 1 h, after which it was removed and replaced with HPLM containing 100 mg/mL gentamicin for the remainder of the experiment. To confirm the efficacy of gentamicin bacterial killing, *E. faecalis* bacteria were cultured in HPLM media alone for 6 h, followed by the addition of 200 mg/mL gentamicin. At 7 h, the samples were either left as is or the media was removed and replaced with 100 mg/mL gentamicin in media. Supernatant was removed and serially diluted from all wells at 6, 7, and 24 h and plated out on BHI agar for CFU enumeration.

### Transmission electron microscopy

Cultures of neutrophils with and without bacteria were fixed for 1 h in 2.5% glutaraldehyde (Ted Pella, Inc.) in PBS, pH 7.3, washed in buffer, post-fixed for 1 h in 1% osmium tetroxide with 1.5% potassium ferrocyanide in PBS, then dehydrated in an ethanol series followed by absolute acetone and embedded in Araldite 502 resin (Ted Pella, Inc). Ultra-sections were cut at 100 nm on a Leica EM UC6 ultramicrotome, collected on 200-mesh copper grids covered with a Formvar-carbon support film (Electron Microscopy Sciences), and stained for 8 min in lead citrate stain. Photographs were taken with Transmission Electron Microscopy (JEM-1400Flash, JEOL Ltd.) at 100 kV with a bottom-mounted, high-sensitivity sCMOS camera.

### Multiplex cytokine analysis

Following infection of murine neutrophils with MOI 1 of *E. faecalis* OG1RF above, or stimulation with 10 mg/mL *S*. *pyogenes* lipoteichoic acid (Sigma Aldrich), cell culture supernatants were taken at 6 h and 24 h p.i. These were prepared according to the manufacturer’s instructions and loaded onto a Bio-Plex PRO^TM^ Mouse Cytokine 23-plex Assay (Bio-Rad, California) to evaluate total cytokine concentrations. Sample processing, plate analysis, and calculation of cytokine concentrations were performed at Bio-Rad Laboratories, Singapore, with the assistance of their technical team.

### Statistical analysis

Statistical analyses were performed utilizing GraphPad Prism software (Version 10). Figure legends list specific test employed. For data sets involving two groups, an unpaired *t*-test was used. For data sets involving three or more groups, a one-way ANOVA with multiple comparisons test was performed. Details on specific tests applied and data obtained are contained within figure legends.

## SUPPLEMENTAL MATERIAL

Supplemental material iai.00364-25-s0001.pdf Fig. S1 to S4.

ASM does not own the copyrights to Supplemental Material that may be linked to, or accessed through, an article. The authors have granted ASM a non-exclusive, world-wide license to publish the Supplemental Material files. Please contact the corresponding author directly for reuse.
