# Supplementary material for: Enterococcus faecalis persists and replicates intracellularly within neutrophils
Source: Infect Immun. 2025 Dec 16;94(1):e00364-25. doi: 10.1128/iai.00364-25 (PMC12797935; doi:10.1128/iai.00364-25)
Supplement: Supplemental material — Fig. S1 to S4. [file iai.00364-25-s0001.pdf]

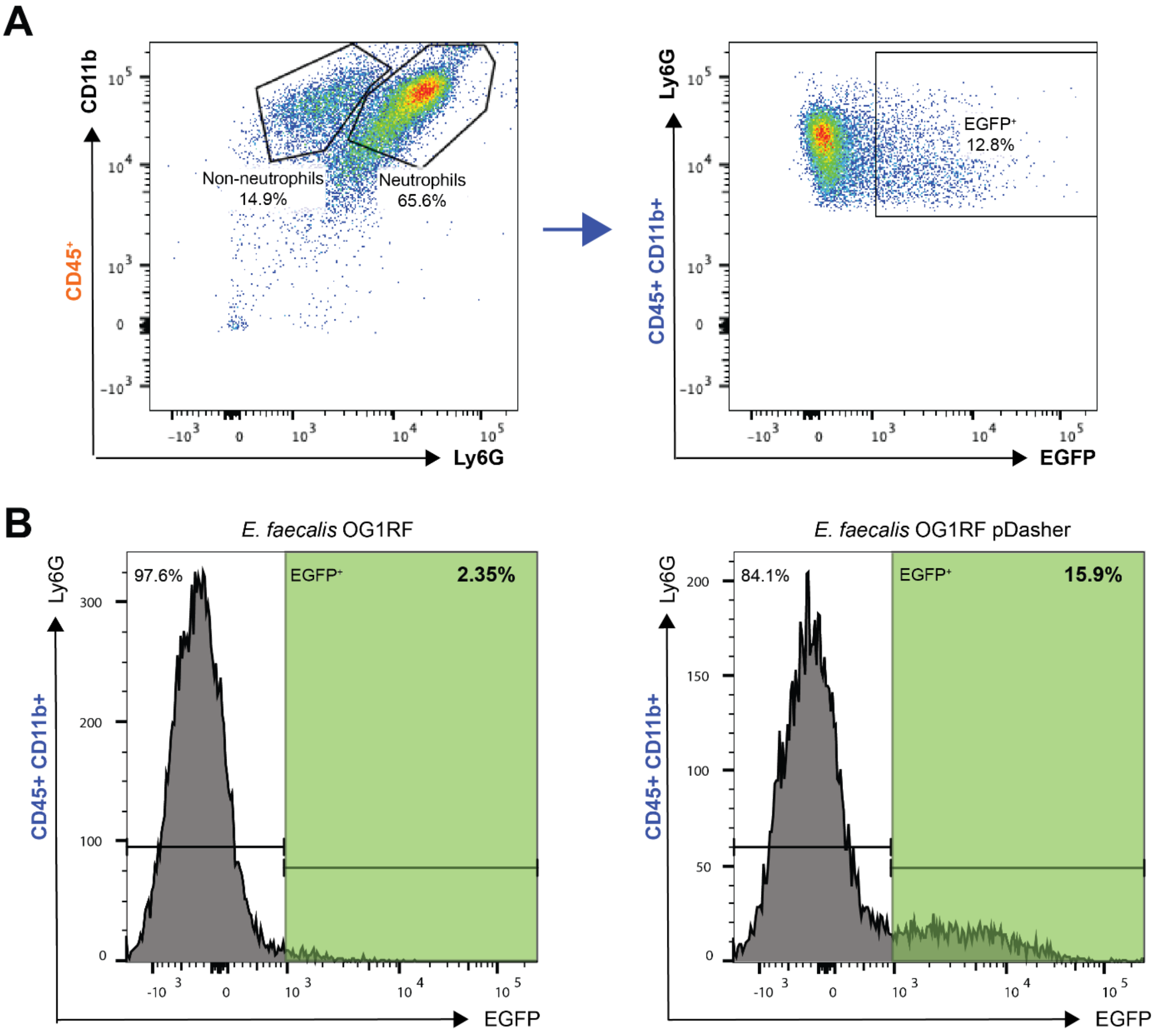

Supplementary Figure 1: *E. faecalis* intracellular clusters can be detected within neutrophils. OG1RF pDasher infected mouse wounds were harvested at 24 h p.i. **(A)** Representative flow cytometry panel of neutrophils (CD45^{+} CD11b^{+} Ly6G^{high}) and non-neutrophils (CD45^{+} CD11b^{+} Ly6G^{low}) from infected wounds. **(B)** Representative staining profiles of wound infected with wildtype OG1RF (EGFP-) and OG1RF pDasher (EGFP+) indicating EGFP positive gating.

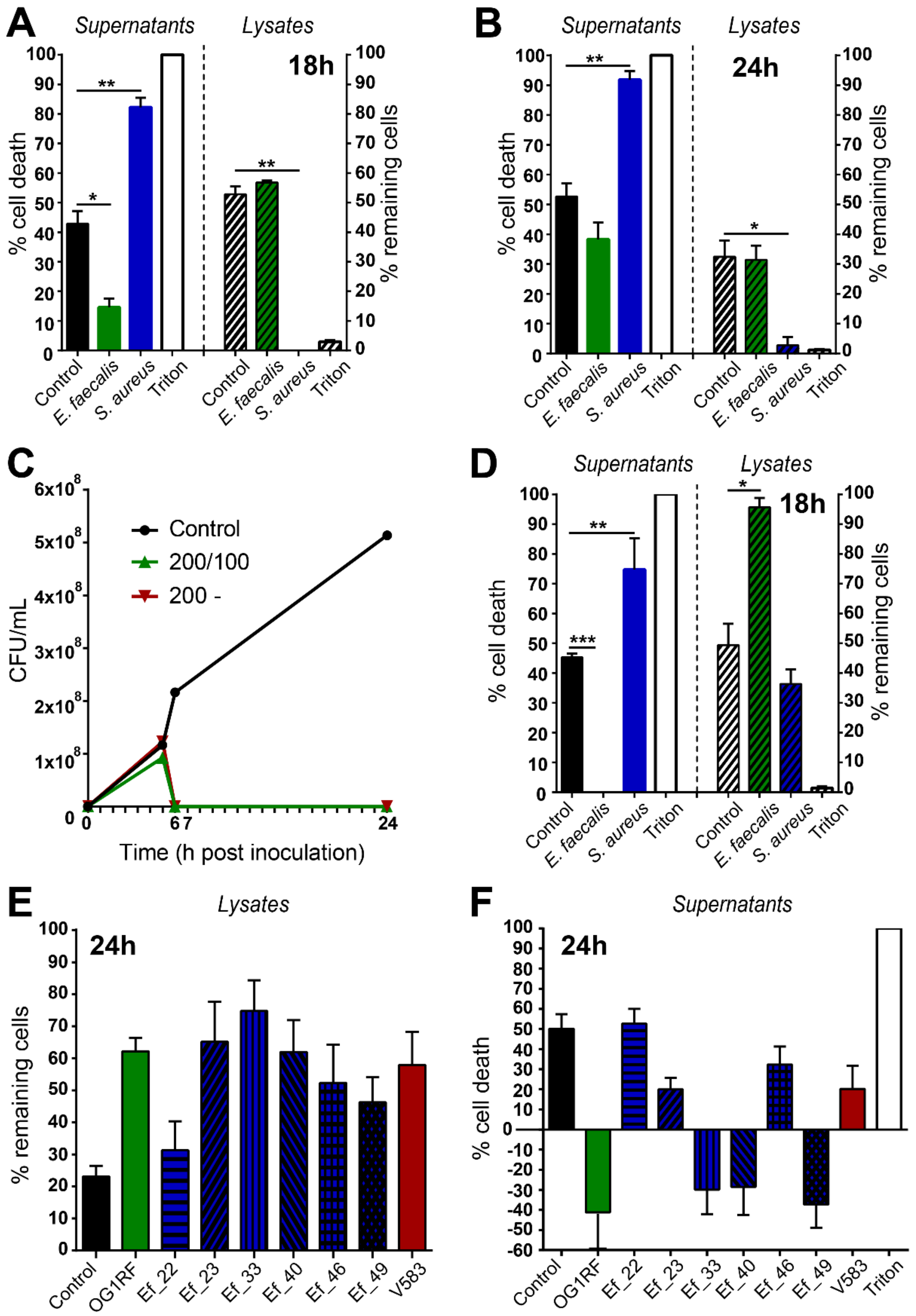

Supplementary Figure 2: Infection with *E. faecalis* suppresses spontaneous neutrophil cell death, in contrast to *S. aureus*. (A-B, D-F) Murine neutrophils were infected with *E. faecalis* OG1RF or *S. aureus* USA300 (MOI 1) for 6 h, followed by gentamicin exclusion of extracellular bacteria.. At the indicated timepoint, LDH assay was performed on supernatants and remaining cells lysed identically in media. Total percentage cell death in both cases was calculated by comparison to 100% kill well 'Triton'. **(C)** *E. faecalis* bacteria were cultured in HPLM media for 6 h, followed by the addition of 200 µg/mL gentamicin. At 7 h this was left as in, or the media removed and replaced with 100 µg/mL gentamicin in media. Supernatant

was removed and serially diluted from all wells at 6, 7, and 24 h, before being plated on BHI agar for CFU enumeration. **(A-B, D-F)** Data depicts (n=3, 4, 4, 3,3) experiments, respectively, mean+SEM. Data was analysed using One Way ANOVA and Dunnett's multiple comparisons test, * denotes $p<0.05$, ** $p<0.01$, ***$p<0.001$. **(C)** Is representative of n=2 experiments.

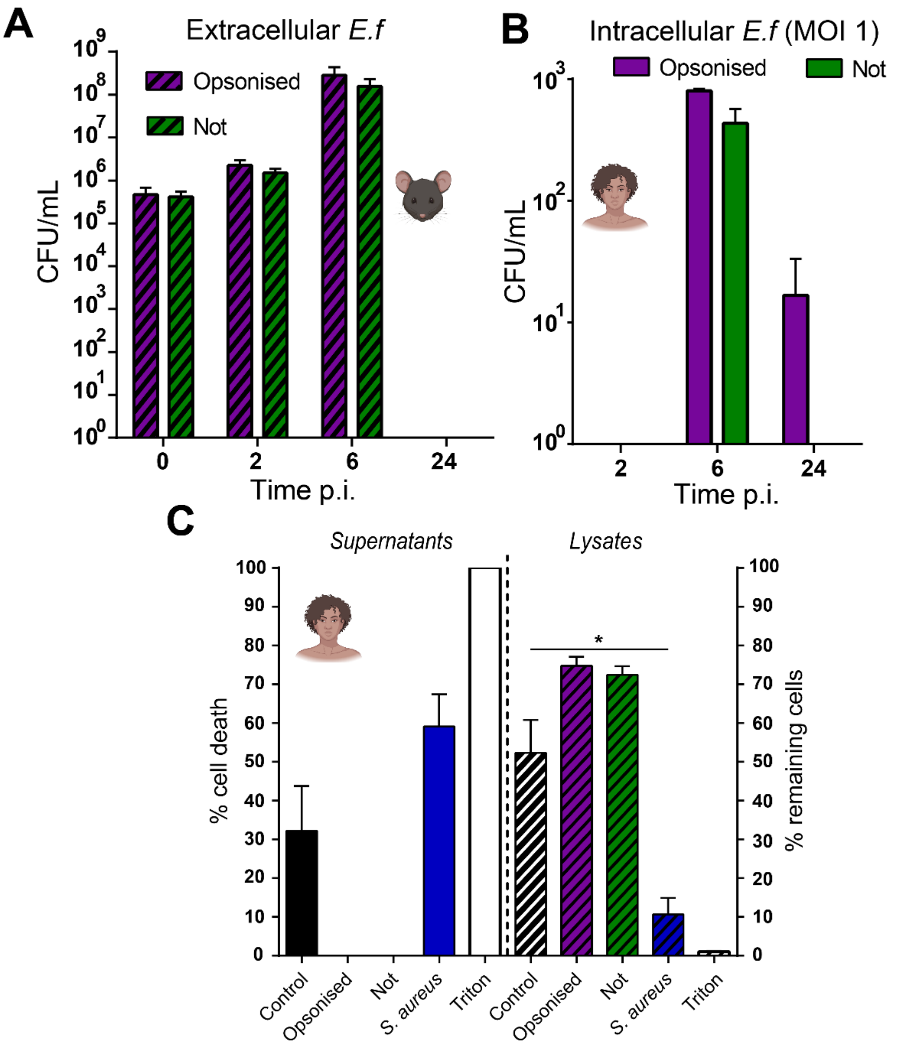

Supplementary Figure 3: *E. faecalis* is engulfed by neutrophils and persists intracellularly out to 24 h p.i. Murine **(A)** or human **(B, C)** neutrophils were infected with **(A, B)** MOI 1 or **(C)** MOI 10 *E. faecalis* OG1RF. The infection was halted at either 2 h (for 2 h timepoint) or 6 h (6 and 24 h timepoints) after which extracellular bacteria were killed by gentamicin exclusion. **(A)** At the given timepoints, a sample of supernatant was taken, or **(B)** wells were gently washed once with PBS, then lysed in PBS + 0.1% triton and serially diluted onto BHI agar for CFU enumeration. **(C)** LDH assay was performed on supernatants and remaining cells lysed identically in media at 24 h p.i. Total percentage cell death in both cases was calculated by comparison to 100% kill well 'Triton'. Data depicts mean + SEM, from (n=4, 3, 4) independent experiments, respectively. **(C)** Data was analyse using One Way ANOVA with Dunnett's multiple comparisons test, * denotes $p<0.05$.

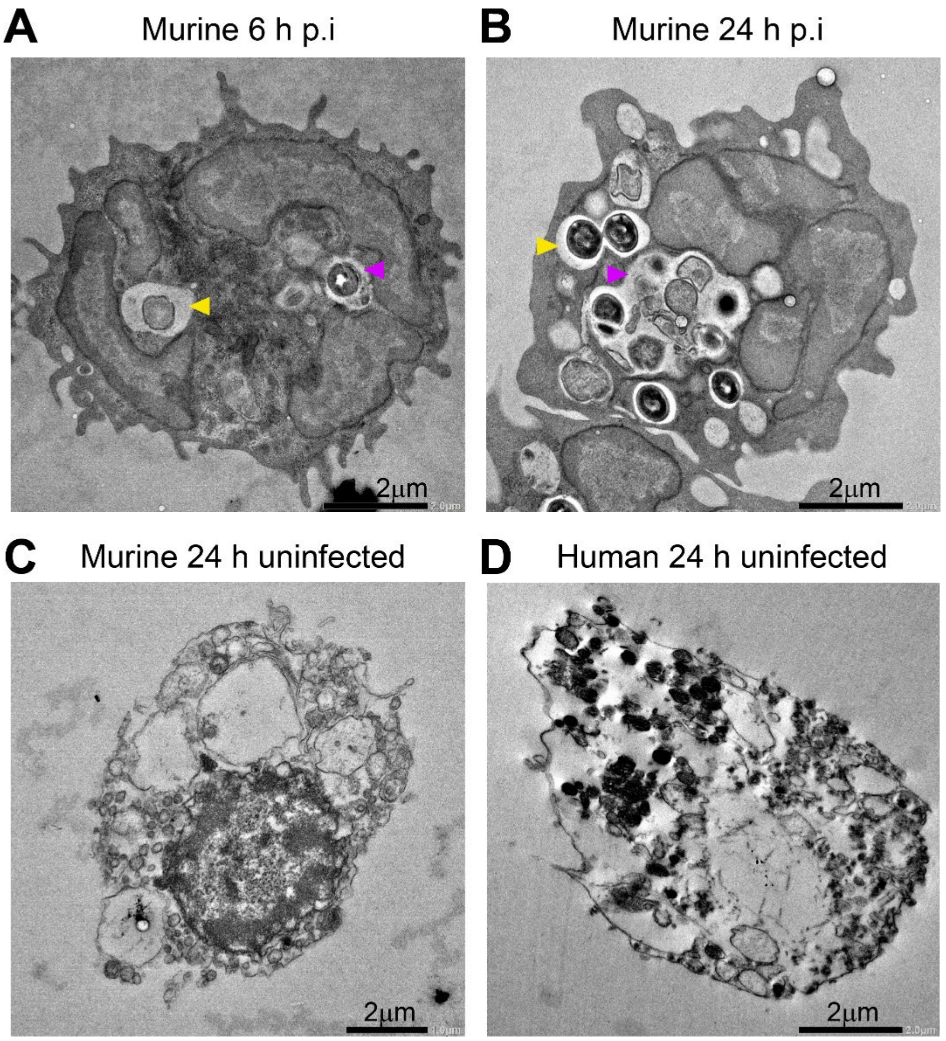

Supplementary Figure 4: *E. faecalis* resides within membrane-bound vacuoles within murine and human neutrophils. (A-B) Murine neutrophils were infected with MOI 1 of *E. faecalis* OG1RF for 6 h, followed by gentamicin exclusion of extracellular bacteria. Murine **(C)** or human **(D) n**eutrophils were left uninfected. At **(A)** 6 h or **(B-D)** 24 h p.i., cells were washed with PBS then fixed with 2.5% glutaraldehyde. Cells were imaged via transmission electron microscopy. Depict individual cells from their respective timepoints, representative of at least 6 cells / condition. Yellow arrowheads mark larger, intact membrane bound compartments, with magenta arrowheads a vacuole with a partial/degraded membrane.
